# Geochemical Characteristics and Ecological Risk Assessment of Heavy Metals in Surface Soil of Gaomi City

**DOI:** 10.3390/ijerph18168329

**Published:** 2021-08-06

**Authors:** Zongjun Gao, Hongzhi Dong, Songtao Wang, Yuqi Zhang, Hairui Zhang, Bing Jiang, Yang Liu

**Affiliations:** 1 College of Earth Science and Engineering, Shandong University of Science and Technology, Qingdao 266590, China; zongjungao1964@163.com (Z.G.); dhz@sdust.edu.cn (H.D.); 2 The Fourth Geological Brigade of Shandong Provincial Bureau of Geology and Mineral Resources, Weifang 261021, China; wangsongtao@sddksy.com (S.W.); zhanghairui@sddksy.com (H.Z.); jiangbing@sddksy.com (B.J.); liuyang@sddksy.com (Y.L.); 3 Key Laboratory of Coastal Zone Geological Environment Protection of Shandong Geology and Mineral Exploration and Development Bureau, Weifang 261021, China

**Keywords:** surface soil, heavy metals, geochemical characteristics, ecological risk assessment, Gaomi City

## Abstract

Gaomi City, the hinterland of Jiaolai Plain in Shandong Peninsula, was selected as the research object. A total of 8197 surface soil samples were collected to determine the contents of eight soil heavy metals (HMs)including Copper (Cu), Lead (Pb), Zinc (Zn), Nickel (Ni), Chromium (Cr), Cadmium (Cd), Arsenic (As), and Mercury (Hg). Statistical methods were used to find out the geochemical background (GCB) in the area, systematic clustering and factor analysis were used to study the homology between HMs, and single-factor evaluation method was used to evaluate the ecological risks in the study area. The results of the study show that the ecological risk of the surface soil in the study area is relatively low, dominated by a planar distribution, with only a few high-risk points. The uneven distribution of Hg in the surface soil is affected by human activities to a certain extent. The ratio of the GCB of the geological unit area to the GCB of the whole area shows that the Hg content of the Qingshan Group and Dasheng Group geological units is higher, and the Pb content in the subvolcanic rock area is slightly higher. The ecological pollution risk in the study area is generally low, and only exists individual high-risk areas, distributed radially in densely populated areas.

## 1. Introduction

Previous studies have shown that human activities have a strong disturbance effect on the distribution of HMs [1,2]. With the continuous use of chemical fertilizer, not only is the content of HMs in soil increasing, but the bioavailability of HMs in soil is also changing [3,4,5,6,7]. When the accumulation of HMs in soil exceeds the soil capacity, crop production will be reduced and the sustainable development of agriculture will be directly restricted [8,9]. Meanwhile, with the rapid development of the economy, and the acceleration of industrialization, urbanization, and agricultural modernization, the risk of farmland soil being polluted is increasing [10,11]. Studies have shown that land-use changes due to urban sprawl result in rising levels of impervious cover, which affects the vertical and vertical migration law of HMs [12]. Heavy metal elements in surface soil are not only controlled by human activities, but also affected by geological background, which can be distinguished according to land use types [13,14]. Rock is broken by weathering to form loose debris, called soil parent material, of which physical and chemical properties change, is the basic primitive material of soil. Previous studies have found that the soil parent material is an important natural source of HMs, which determines the initial heavy metal content in the soil [15,16,17]. In general, different geological units contain different types of soils. Indeed, even one individual geological unit may contain multiple types of soils. Soil heavy metal pollution is characterized by concealment, irreversibility, and long-term nature, which increases the difficulty of heavy metal pollution control [18]. HMs in the environment, soil, and water can eventually find their way into the human body by way of food chain, and therefore people consume heavy metal elements through diet every day [19,20].

With the increasing use of land by human beings, soil environmental pollution is becoming more and more serious, especially heavy metal pollution in soil. It is necessary to study the GCB in the geological-unit perspective and in different types of land use areas.

Clear waters and green mountains are as good as mountains of gold and silver. Development at the expense of the environment has come to an end, and harmonious coexistence with the environment is the ideal long-term solution. From traditional agriculture to modern agriculture to the emergence of green agriculture, ecological agriculture and organic agriculture, soil quality is the key factor restricting agricultural transformation. Therefore, determining soil quality and heavy metal geochemical characteristics of agricultural land and assessing its ecological risk are prerequisites for effective land conservation and utilization. It is urgent to deal with the polluted cultivated land, protect the unpolluted cultivated land and develop different farming methods according to the condition of the land. The parameters of GCB are basic characteristic parameters in soil geochemical investigation and research, which represent the content level and changing rules of elements in soil [21].

Gaomi City is rich in geochemical and hydrogeochemical research foundation, but it mainly focuses on the source, migration, and enrichment law of fluorine in groundwater. However, as an important vegetable and grain planting base in Shandong Province, there are few investigations and studies on the distribution law of HMs in soil [22,23]. In this study, soil in Gaomi City is taken as the studied object, and the contents of eight types of HMs, including Cu, Pb, Zn, Ni, Cr, Cd, As, and Hg, were tested, which are the necessary items for screening the risk of soil pollution on agricultural land [24,25]. Based on the test data, geochemical characteristics of HMs in soil of Gaomi were analyzed and ecological risk assessment was carried out according to China national standards of environmental quality standard for soils [24,25]. The purpose of this work is to find out the geochemical characteristics and ecological risk of HMs in surface soil of Gaomi City, so as to ensure the safety of agricultural products and formulate more reasonable plans for agricultural production and fertilizer use. The background values of heavy metal elements in the topsoil of the whole area, geological unit area, and land use type area were respectively studied.

## 2. Research Background

**Figure 1:**
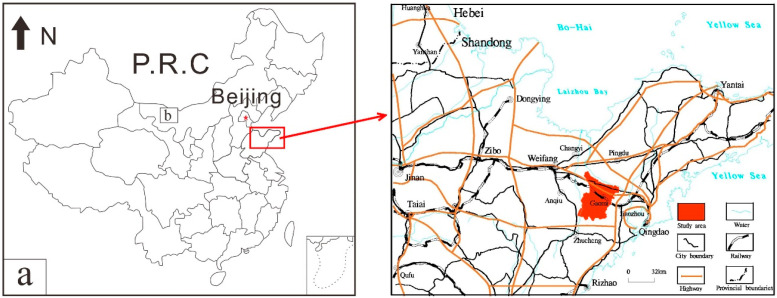
Location map of study area (**a**) Location of Shandong Peninsula (**b**) Location of study area.

Gaomi City is located in Weifang City, Shandong Province, in the central part of Shandong Peninsula and the hinterland of Jiaolai Plain (Figure 1). Due to oscillating-upward crustal movement in the long geological history period, the rocks were exposed to weathering and denudation for a long time, forming the landform of low-lying hills and peneplain [22]. The main geological units include Mesozoic Laiyang group, Qingshan group, Latent volcanic rocks and Dasheng group; Cenozoic Dachan formation, Heituhu formation, Linyi formation and Yihe formation. The main rock types are pebbly sandstone, sandstone, siltstone, shale, pyroclastic rock, volcanic lava, conglomerate, and clay rock [23,26]. The Laiyang Group is a complex set of continental variegated clastic rocks with local volcanic rocks and a small amount of volcanic clastic deposits with fluvial-lacustrine facies. The Qingshan Group is a set of complex continental acidic volcanic rocks, intermediate-intermediate basic volcanic rocks, and volcano-sedimentary rocks. The lithology in the subvolcanic unit area is relatively single, which is rhyolite fused breccia tuff and glassy tuff formed by the eruption of acid volcanic magma. The Dasheng Group is a set of continental clastic rocks interbedded with volcanic rocks. The composition of Dazhan Formation belongs to the Middle Pleistocene and the Upper Pleistocene, and the sedimentary facies are eolian and alluvial, respectively. The Heituhu Formation is coastal lacustrine facies. The Dazhan Formation and the Heituhu Formation have different properties due to the different accumulation genesis. Both the Linyi and Yihe Formations are closely related to modern rivers, with the former as floodplain deposits and the latter as modern fluvial facies deposits [27,28,29,30]. In general, the terrain is higher in the south and lower in the north, with a maximum length of 60.1 km from north to south and a maximum width of 51.2 km from east to west, covering an area of 1525.70 km^2^. Gaomi City belongs to the warm temperate continental semi-humid climate in the monsoon area, with cold winter and hot summer, and four distinct seasons. The average annual precipitation is 689.1 mm. The main land use type in the study area is cultivated land, followed by urban and village land. The region is dominated by grain planting, supplemented by vegetable planting. Among them, Xiazhuangdajingou leek is the local leek variety of Gaomi City, which was approved to use the public logo of agricultural product geographical indication in May 2012 [31]. Jiaohe potato is a specialty of Baicheng Town, Gaomi City, and is a product of China’s national geographical indication [32].

## 3. Samples and Methods

### 3.1. Sample Collection

According to the “Specification for Geochemical Evaluation of Land Quality”(DZ/T 0295-2016) [33] and based on the latest 1:50,000 land use status map, the samples were mainly collected from agricultural land, other land samples were arranged according to the minimum requirements of the sampling density range, taking into account the uniform distribution of space. The design sampling unit was 1 km^2^ and the sampling density of soil samples is 4~8 pieces/km^2^, and the actual sampling average control density was 5.5 pieces/km^2^, 8197 samples in total. In order to take into account the uniformity of spatial distribution, during sample collection, the preset sampling position was taken as the center of each sampling unit. Each sample consists of 4–6 samples mixed in equal quantities in equal quantities which comes from 1 main sampling point and 3–5 sub-sampling points, and the locations of the sub-sampling points were determined by radiating 50~100 m to the main sampling point.

**Figure 2:**
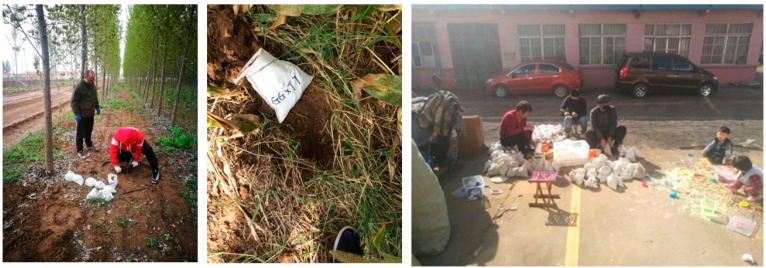
Pictures of sample collection and processing site.

The sampling depth of the topsoil is randomly between 0~20 cm. The soil at each sampling point was crushed, the debris in samples were picked out, and the same amount of sample was mixed into different clean cloth sample bags, not less than 1 kg into an individual bag. All samples were collected and analyzed in the same way within one year in sunny weather. Sample collection and processing site are shown in Figure 2.

### 3.2. Samples Preparation and Testing

The collected topsoil samples were hung in a cool place and naturally air-dried on the sample rack. After air-dried, they were spread on the sample making plate, crushed with a wooden stick, and sundries were removed. All the samples were mixed through a nylon sieve with a diameter of 2 mm, weighed, and put into a plastic bottle at least 500 g for transfer to the laboratory for testing. During collection and processing, the samples did not make contact with metal utensils.

The sample preparation and testing were completed by the Experimental Test Center of the Fourth Geological and Mineral Exploration Institute of Shandong Province. The soil samples were processed to 0.074 mm by pollution-free geochemical exploration crushing machine, without shrinkage and screening, and were directly put into sample bags for testing. X-ray fluorescence spectrometry (XRF) was used to detect Cu, Pb, Zn, Ni, and Cr, and inductively coupled plasma mass spectrometry (ICP-MS) was used to detect Cd. As was detected by atomic fluorescence spectrometry (AFS), and Hg was detected by steam generation cold atomic fluorescence spectrometry (AFS) [24,25,34]. The detection limit of the analytical method used for each element is Cu (1 × 10^−6^), Pb (2 × 10^−6^), Zn (4 × 10^−6^), Ni (2 × 10^−6^), Cr (5 × 10^−6^), Cd (0.02 × 10^−6^), As (0.5 × 10^−6^), Hg (0.5 × 10^−9^).

In order to ensure the accuracy and precision of experimental data, both internal quality control (IQC) and external quality control (EQC) were adopted. According to the “Technical Requirements for Analysis of Ecological Geochemical Evaluation Samples” (DD2005-03) [35], in the process of IQC 4 national first-level reference materials (GBW) of certified reference materials(CRM) were inserted for every 50 samples. In the process of EQC 2 external standard control samples of reference materials (RM) were inserted for every 50 samples. According to the statistical results of accuracy and precision of total analysis of soil elements, the qualified rate of accuracy and precision is 100%, which meets test quality requirements of the “Specification of Land Quality Geochemical Assessment” (DZ/T 0295-2016) [33].

### 3.3. Data Processing and Mapping

Excel is used to eliminate the ultra-high and low values by iterative method, and the average value of element content is obtained to determine GCB. The systematic cluster analysis and factor analysis of 8 types of heavy metal elements in the topsoil of the study area were carried out by SPSS22.0. Spatial distribution mapping was carried out by universal Kriging method in MapGIS6.7 [36].

## 4. Results-Geochemical Characteristics of HMs in Topsoil

### 4.1. GCB of HMs in Topsoil

#### 4.1.1. GCB of Heavy Metal Elements in the Topsoil of the Whole Region

The statistics of heavy metal element GCB in the topsoil are shown in Table 1. Among the eight HMs, only the variation coefficients of Pb and Cr were less than 0.3, showing a relatively uniform distribution. The variation coefficients of other heavy metal elements are all greater than 0.3, showing a relatively obvious spatial distribution difference, among which the variation coefficient of Hg is the highest as 4.13, indicating that the spatial distribution difference of Hg element in the topsoil is extremely significant. In addition, by comparing the GCB of heavy metal elements in the topsoil of the study area with those in Weifang City [37] and Shandong Province [38], the GCB ratios (GCBRs) can be calculated. It is found that: (1) The GCBRs of HMs in the surface soil of the study area to those in Weifang City and Shandong Province are between 0.77~1.12, 0.72~1.02, respectively, indicating that the content of heavy metal elements in the topsoil of the study area is basically consistent with the average level of Weifang City and Shandong Province. (2) Six of the eight heavy metal elements in the study area were lower than GCB of Weifang and Shandong Province, Pb was similar to the GCB of Weifang and Shandong Province, and only As was slightly higher than that of Weifang. Comparing the median of 8 HMs with the maximum allowable limits of heavy metal in soils of WHO and China (Table 2). It is demonstrated that the content of HMs in the topsoil of the whole region is under the limits.

#### 4.1.2. GCB of Heavy Metal Elements in Surface Soil of Different Geological Unit Areas

**Figure 3:**
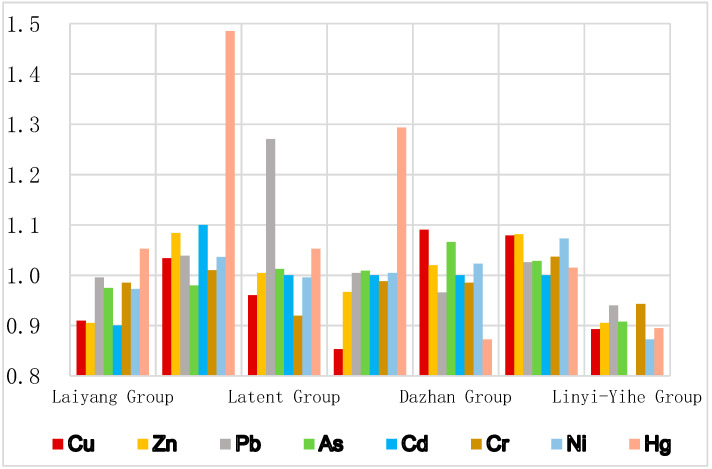
Bar chart of GCBRs.

A total of seven geological units were divided, including Laiyang Formation, Qiangshan Formation, Qianhuoshan Formation, Dasheng Formation, Dazhan Formation, Heituhu Formation, and Linyi-Yihe Formation. Areas of each formation are shown in Table 3. The soil GCB of 7 types of geological units were listed in Table 4. Soil GCBRs between each geological unit with the whole area could also be found in Table 4. A visual comparison of GCB and GCBRs of each geological unit are shown in Figure 3.

As shown in Table 4 and Figure 3. Through analysis, it is found that the heavy metal elements in the topsoil of Laiyang Group are close to the background values of the whole region, with the GCBRs of 0.90~1.05. The ratio of Hg to the background values of the whole region is relatively high (GCBRs 1.48), which is the main distribution area of most heavy metal anomalies. Qingshan Group, except for As (GCBRs 0.98), is relatively lower than others. Moreover, excluding the GCBRs of Pb (1.27) as obviously higher, the other elements in this geological unit are close to the background value of the whole area (GCBRs 0.92~1.05), which is the main distribution area of Pb anomaly. In Dasheng Group, except Cu (GCBRs 0.85) and Hg (GCBRs 1.29), other elements are close to the background values of the whole region (0.97~1.01). Due to the differences of accumulation origin and properties of Dazhan Formation and Heituhu Formation, it is found that, except Hg in the Dazhan Formation, which is lower than the background value of the whole area (GCBRs 0.87), the heavy metal elements in the topsoil in these two units are close to and slightly higher than the background values of the whole area, especially higher than the background value of the whole area in the Heituhu Formation. Compared with the background values of the whole region, the heavy metal elements in the topsoil of Linyi and Yihe Formations are lower or even significantly lower, which is the main distribution area of negative anomaly, indicating that there is no heavy metal pollution source at the source and banks of the river. Based on the comprehensive analysis of the whole region, it is found that the background values of heavy metal elements in different geological unit areas have a good correlation with the background values of the whole region, and only show anisotropy at a few points, indicating that the content of heavy metal elements in the topsoil of the whole region is mainly affected by the geological background.

#### 4.1.3. GCB of Heavy Metal Elements in the Surface Soil of Different Types of Land Use Areas

**Figure 4:**
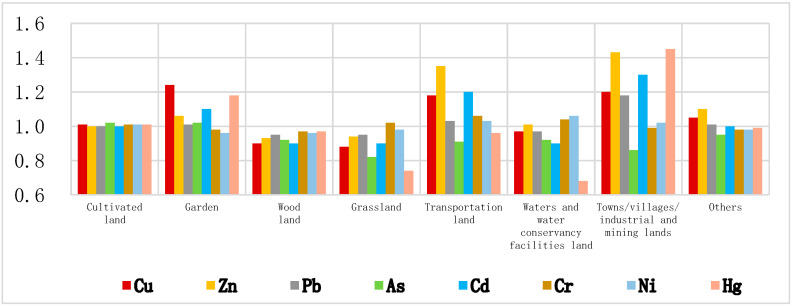
Bar chart of GCBRs.

By studying the distribution of HMs in different types of land, we can analyze the influence of different human activities on the distribution of HMs. According to the land survey results of Weifang City in 2015, the land use types in the study area were divided into eight types and their soil GCBs were statistically analyzed and compared with the GCB of the whole area (Table 5 and Figure 4). Soil GCB ratios between each type of land use area with the whole area (GCBRs) could also be found in Table 5. A visual comparison of GCB and GCBRs of each geological unit is shown in Figure 4.

The land use type in the study area is mainly cultivated land, accounting for 70.90% of the total area. The background value of HMs in the surface soil of cultivated land is very close to the background value of the whole area, indicating that agricultural activities in the study area have a weak influence on the changes of heavy metal contents in the topsoil. The area of the garden occupies 12.00% of the whole area. Cu, Hg, and Cd in this type of area are significantly higher than the background values of the whole area, which are 1.24, 1.18 and 1.10, respectively. Other elements are close to the background values of the whole area (the GCBRs is 0.96~1.06). The results indicate that the agricultural production activities in the garden had a slightly more significant effect on the content of HMs in the topsoil than that in the cultivated land. The background values of most heavy metal elements in woodland, grassland, water area, and water conservancy facilities land are lower than the background values of the whole area, and a few heavy metal elements are close to or slightly higher than the background values of the whole area, which reflects that these land use types are less affected by human disturbance. In the transportation land, Zn, Cu, and Cd are significantly higher than the background values of the whole area, while Pb, Cr, and Ni are slightly higher than the background values of the whole area. These anomalies are mainly distributed on both sides of the highway, and may even form pollution zones. Cu, Pb, Zn, Cd, and Hg in towns, villages and industrial and mining land were significantly higher than the background values of the whole area, while Ni was slightly higher than the background values of the whole area.

Therefore, it can be seen that human activities are an important factor affecting the change of the content of HMs in topsoil, especially the industry, mining industry and transportation industry. In transportation, automobile exhaust and tire wear may produce dust with HMs, which enters the soil through settling, increasing the content of HMs in the topsoil and forming anomalies [14,40].

### 4.2. Homologous-Cluster Analysis of Heavy Metal Elements

#### 4.2.1. Cluster Analysis

**Figure 5:**
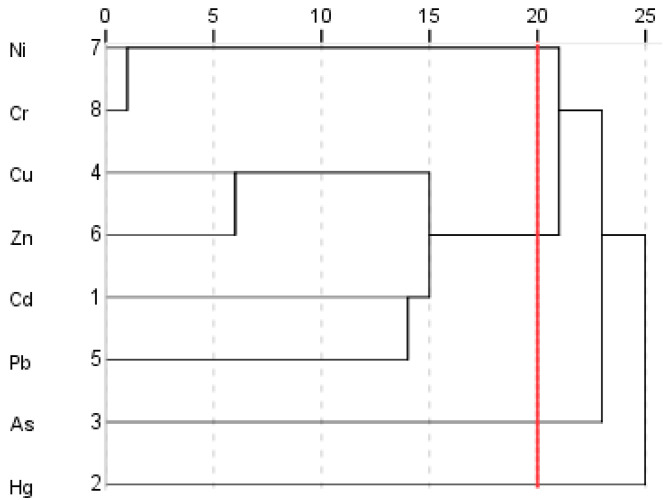
Cluster spectrum of HMs.

In the cluster analysis, elements with symbiotic or similar genesis should have sufficient similarity or homogeneity, while elements with large differences or dissimilar genesis should have great heterogeneity [41]. The heavy metal elements in root soil samples were classified according to similarity degree by system clustering method, and the possible symbiotic relationship or genetic relationship among these heavy metal elements were analyzed. As shown in Figure 5, when the confidence level is five, the eight HMs can be divided into seven categories. Then, only nickel and cadmium are divided into the same group, and other HMs form a group respectively. When the confidence is 10, the eight HMs can be divided into six categories. The first category is Ni and Cr, the second category is Cu and Zn, and other four HMs form a different group. When the confidence is 20, the eight heavy metal elements can be divided into four categories. The first category is Ni and Cr, the second category is Cu, Zn, Cd, and Pb, while the third and fourth categories are As and Hg, respectively. The elements in each category are correlated with each other, displaying high homology.

#### 4.2.2. Homologous Analysis

As shown in Table 6, main causes of the eight elements are listed. The dimensionality reduction of heavy metal element data through principal factor analysis can better discover the original information rules and excavate the homology of heavy metal elements.

In order to obtain the main factor, the eigenvalue and corresponding eigenvector of the correlation matrix are calculated, and the cumulative percentage is obtained according to the percentage of eigenvalue (variance contribution). The factors with a large eigenvalue and cumulative contribution rate of more than 80% are selected as the main factors. According to the eigenvalue calculation results the eight heavy metal elements in the study area can be extracted as three major factors, (Table 7), and the heavy metal elements in each major factor have similar sources or homology [42]. According to the extraction results (Table 8), the three main factors can represent 63.146% of the cumulative contribution of the eight HMs, and the characteristic roots are all greater than 1. That is to say, the three main factors can basically represent the distribution characteristics of the eight HMs. Taking the factor load greater than the constant value of 0.5 as the criterion, the combined characteristics of the eight heavy metal elements in the study area were obtained. The characteristic root percentage of the main factor 1 was 26.641%, and its variables included Cd, Cu, Pb, and Zn. The characteristic root percentage of the main factor 2 was 22.401%, and its variables included Ni and Cr. The characteristic root percentage of the main factor 3 was 14.105%, and its variables included Hg and As. The results of principal factor analysis are basically consistent with those of cluster analysis.

**Table 6:** Main causes of the eight elements.

Elements	Main Causes	References
Cd	Smelting of non-ferrous metals, disposal of cadmium-containing wastes.	[43,44,45,46]
Cu	Smelting emissions soot, industrial coal, automobile exhaust.	
Pb	Gas from gasoline combustion, lead paint, smelting, casting.	
Zn	Smelting, waste incineration, rubber tire wear.	
Ni	Smelting, roasting, automobile exhaust.	
Cr	Metal processing, electroplating, tanning, coal burning, oil burning.	
Hg	Coal-fired power plants, mining and processing of related mineral materials.	
As	Industrial production, use of arsenic-containing pesticides, coal burning.	

**Figure 6:**
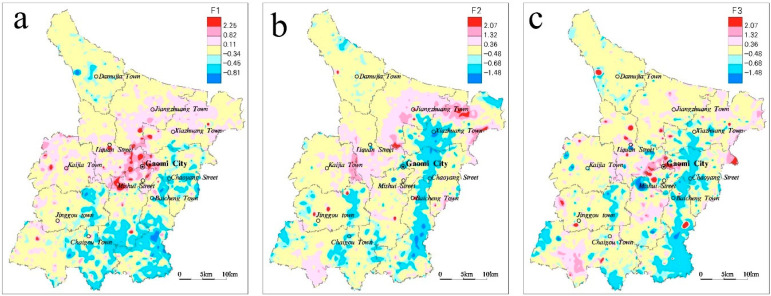
Score contour map for three main factors (**a**) factor 1 (**b**) factor 2 (**c**) factor 3.

The high-score region of main factor 1 (Figure 6a) is mainly distributed in the densely populated and industrially developed areas such as Gaomi urban area and its surrounding towns, indicating that human activities are the main reason for the high content of each element of main factor 1. The Ni and Cr in the main factor 2 belong to the iron group elements, which are difficult to migrate, and are generally attached to the soil parent material in the form of secondary minerals. From the distribution characteristics (Figure 6b), it can be seen that the high-score zone is not quite consistent with the densely populated areas of human activities, while the low-score zone is mainly distributed along the Jiaohe River, Wulong River and the alluvial zones on both sides of the river. This is mainly because the heavy metal elements are carried away by the current and replaced by river sediments under the scouring action of the river. Therefore, it can be concluded that the main factor 2 is affected by the geological background and geomorphic form at the same time. The main factor 3 high-yield area (Figure 6c) mainly presents a point-source distribution, which is relatively concentrated in the Gaomi urban area and its surrounding areas, indicating that it may be significantly affected by human activities.

## 5. Ecological Risk Assessment

### 5.1. Ecological Risk Assessment Methods

Single-factor ecological risk assessment was carried out as shown in Table 9, combining the risk screening values Si and risk control values Gi of eight HMs given in the risk control standard of soil pollution in agricultural land [24] or the risk control standard of soil pollution in construction land [25].

In addition, the method of potential ecological risk index (RI) was also used in this paper to evaluate the ecological risk of HMs. This method includes two concepts, namely the single factor potential ecological risk coefficient *E_i_* and potential ecological risk index RI. The calculation formulas are as follows:

*E_i_* = *T_i_* × (*C_i_*/*C*_0_) (1)

RI = *∑E_i_* (2)

where *T_i_* is the toxicity coefficient of heavy metal *i* (see Table 10), *C_i_* is the content of heavy metal *i* in topsoil, and *C*_0_ is the background value of heavy metal elements in the topsoil of the study area [47,48].

Through calculation, ecological risks are classified according to the standard in Table 11, and the pollution degree is divided into five grades: mild, moderate, strong, strong, and extremely strong.

### 5.2. Ecological Risk Assessment Results

**Figure 7:**
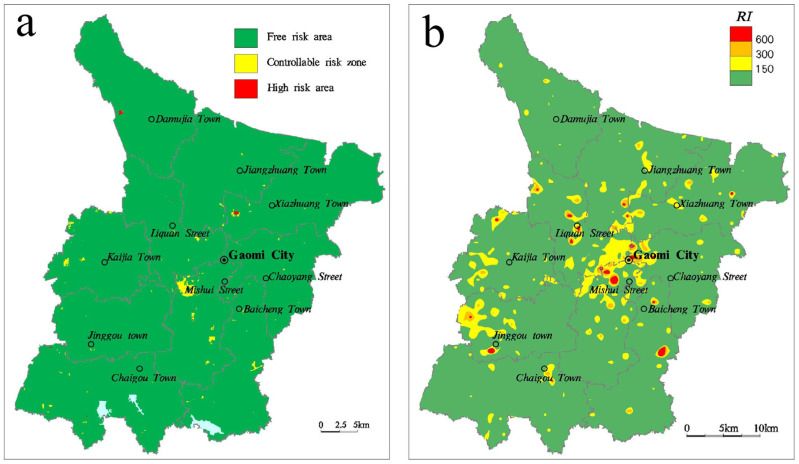
Comprehensive distribution map of study area (**a**) Comprehensive grade map of ecological risk assessment of HMs in topsoil (**b**) Spatial distribution map of Potential risk index of HMs in topsoil.

In the process of the single factor ecological risk assessment, the evaluation unit is drawn according to the sampling unit (1 km^2^). Based on the single factor ecological risk assessment level, the comprehensive ecological risk assessment level of each assessment unit is equal to the worst level of single factor assessment. The evaluation results are shown in Table 12 and Figure 7a. It can be seen that the topsoil in the study area is mainly risk-free. The area of controllable risk is small (9.71 km^2^), which is mainly caused by the excess of the risk screening values of Zn, Cu, Cd, and Ni. However, the area with high risk was only 0.63 km^2^, which was caused by As, Hg, and Cd exceeding the risk control value in a small range.

The calculation results of single factor potential ecological risk coefficient *E_i_* are shown in Table 13. Except for Hg, the average value of other single factor *E_i_* is lower than 40, indicating a low degree of potential ecological pollution risk of single factor. The sum of EI is calculated to obtain the potential ecological risk index RI, and top draw the spatial distribution map of the potential ecological risk index by combining the sampling points (see Figure 7b). It was found that the area with RI value lower than 150 was 1386.44 km^2^, accounting for 90.87% of the total area, and the ecological pollution risk was low. The area with moderate pollution is 117.61 km^2^, accounting for 7.71%, which is mainly distributed around Gaomi urban area and some densely populated areas in towns and villages. The areas with high and strong pollution levels were 15.70 km^2^ and 5.95 km^2^, accounting for 1.03% and 0.39%, respectively. They were distributed in densely populated areas as point sources, mainly caused by the high point values of Hg and Cd. The point source pollution needs to be paid attention to.

## 6. Conclusions

In the Gaomi City, statistical methods were used to find out the geochemical background values, systematic clustering and factor analysis were used to study the homology between heavy metal ions, and single-factor evaluation methods were used to evaluate the ecological risks in the study area. Several certain conclusions were obtained as follows.

(1) Compared with the GCB of Weifang, As is higher, Pb is similar, and others are lower. Compared with the GCB Shandong Province, Pb, As, and Cr are similar, whereas others are lower. The GCB of different geological units and land use types are different, indicating that the distribution of heavy metal elements is affected by geological background and human activities.
(2) The homology cluster analysis showed that the Cd, Cu, Pb, and Zn in the topsoil had similar origin or homology, which were mainly affected by human activities. Ni and Cr have similar origin or homology, and are affected by geological background, geomorphologic form and human activities. Hg and As had similar origin or homology, and the high value area showed radial distribution, which may be related to industrial and mining enterprises.
(3) According to the evaluation of ecological risk based on soil risk management and control standards, the study area is dominated by riskless soils, but there are also some soils with high risks. The average potential ecological risk coefficient of Hg reached moderate pollution degree. Based on an evaluation of potential ecological risk index, the surface soil in the study area is found to contain light pollution, with an area of more than 90%. Meanwhile, there are also moderate pollution, strong pollution, and very strong pollution, among which strong pollution and very strong pollution areas are small, displaying a point-source distribution located in densely populated areas.

## Figures and Tables

**Table 1:** Table of heavy metal element geochemical parameters of surface soil in the study area.

Element	Median/10^−6^	Variation Coefficient	Variation Range/10^−6^	GCB/10^−6^			GCBRs	
				Gaomi	Weifang	Shandong	Gaomi/Weifang	Gaomi/Shandong
Cu	17.4	0.98	7.1–28.3	17.7	21.2	22.6	0.83	0.78
Zn	44.3	0.68	24.3–66.3	45.3	58.5	63.3	0.77	0.72
Pb	23.3	0.23	16.6–30	23.3	22.9	23.6	1.02	0.99
As	8.79	0.49	4.72–12.79	8.75	7.8	8.6	1.12	1.02
Cd	0.10	0.78	0.05–0.15	0.10	0.114	0.132	0.88	0.76
Cr	58.9	0.25	40.3–79	59.6	65.3	62	0.91	0.96
Ni	21.7	0.34	12.1–31.7	21.9	26.9	27.1	0.81	0.81
Hg	0.0252	4.13	0.01–0.05	0.0266	0.032	0.031	0.83	0.86

**Table 2:** Maximum Allowable Limits of Heavy Metal in Soils (10^−6^).

Organization/Country	Cu	Zn	Pb	As	Cd	Cr	Ni	Hg
WHO [20,39]	100	300	100	20	3	100	50	−8
China [24,25]	200	300	240	20	0.8	350	190	1
Median	17.4	44.3	23.3	8.79	0.10	58.9	21.7	0.0252

**Table 3:** Statistical table of geological unit division in the study area.

Geological Unit	Laiyang Group	Qingshan Group	Latent Volcanic Rocks	Dasheng Group	Dazhan Formation	Heituhu Formation	Linyi-Yihe Formation
Area (km^2^)	423.6	11.8	8.50	21.8	185.0	647.0	227.9
Percentage (%)	27.76	0.78	0.56	1.43	12.13	42.41	14.94

**Table 4:** Statistical table of soil GCBs and GCBRs of each geological unit area.

Geological Unit		Elements							
		Cu	Zn	Pb	As	Cd	Cr	Ni	Hg
Laiyang Group	GCB/10^−6^	16.10	41.00	23.20	8.53	0.09	58.70	21.30	0.028
	GCBRs	0.91	0.91	1.00	0.97	0.90	0.98	0.97	1.05
Qingshan Group	GCB/10^−6^	18.30	49.10	24.20	8.57	0.11	60.20	22.70	0.040
	GCBRs	1.03	1.08	1.04	0.98	1.10	1.01	1.04	1.48
Latent volcanic rocks	GCB/10^−6^	17.00	45.50	29.60	8.86	0.10	54.80	21.80	0.028
	GCBRs	0.96	1.00	1.27	1.01	1.00	0.92	1.00	1.05
Dasheng Group	GCB/10^−6^	15.10	43.80	23.40	8.83	0.10	58.90	22.00	0.034
	GCBRs	0.85	0.97	1.00	1.01	1.00	0.99	1.00	1.29
Dazhan Formation	GCB/10^−6^	19.30	46.20	22.50	9.33	0.10	58.70	22.40	0.023
	GCBRs	1.09	1.02	0.97	1.07	1.00	0.98	1.02	0.87
Heituhu Formation	GCB/10^−6^	19.10	49.00	23.90	9.00	0.10	61.80	23.50	0.027
	GCBRs	1.08	1.08	1.03	1.03	1.00	1.04	1.07	1.02
Linyi-Yihe Formation	GCB/10^−6^	15.80	41.00	21.90	7.94	0.08	56.20	19.10	0.024
	GCBRs	0.89	0.91	0.94	0.91	0.80	0.94	0.87	0.89

**Table 5:** Statistical table of soil GCBs in different types of land use areas.

Land Use Types		Elements							
		Cu	Zn	Pb	As	Cd	Cr	Ni	Hg
cultivated land	GCB/10^−6^	17.8	45.3	23.4	8.93	0.1	59.9	22.1	0.0268
	GCBRs	1.01	1.00	1.00	1.02	1.00	1.01	1.01	1.01
Garden	GCB/10^−6^	21.9	47.8	23.5	8.94	0.11	58.5	21	0.0313
	GCBRs	1.24	1.06	1.01	1.02	1.10	0.98	0.96	1.18
Woodland	GCB/10^−6^	16	42.1	22.2	8.06	0.09	58	21	0.0258
	GCBRs	0.90	0.93	0.95	0.92	0.90	0.97	0.96	0.97
Grass land	GCB/10^−6^	15.6	42.6	22.1	7.17	0.09	61	21.5	0.0197
	GCBRs	0.88	0.94	0.95	0.82	0.90	1.02	0.98	0.74
Transportation land	GCB/10^−6^	20.8	61.1	24.1	7.97	0.12	63.1	22.5	0.0256
	GCBRs	1.18	1.35	1.03	0.91	1.20	1.06	1.03	0.96
Waters and water conservancy facilities land	GCB/10^−6^	17.2	45.7	22.5	8.08	0.09	62	23.2	0.018
	GCBRs	0.97	1.01	0.97	0.92	0.90	1.04	1.06	0.68
Towns, villages and industrial and mining lands	GCB/10^−6^	21.3	64.6	27.4	7.52	0.13	59.3	22.3	0.0387
	GCBRs	1.20	1.43	1.18	0.86	1.30	0.99	1.02	1.45
Others	GCB/10^−6^	18.5	49.9	23.5	8.3	0.1	58.4	21.4	0.0263
	GCBRs	1.05	1.10	1.01	0.95	1.00	0.98	0.98	0.99

**Table 7:** Common factor eigenvalue calculation.

Variable	Initial Value	Cumulative Characteristic Root%	Variable	Initial Value	Cumulative Characteristic Root%
Cd	1.000	0.628	Ni	1.000	0.840
Cu	1.000	0.771	Cr	1.000	0.741
Pb	1.000	0.654	Hg	1.000	0.989
Zn	1.000	0.793	As	1.000	0.970

**Table 8:** Heavy metal main factor loading matrix and factor extraction results in the study area.

Variable	Main Factor 1	Main Factor 2	Main Factor 3
Cd	0.652	−0.007	0.289
Hg	0.089	−0.137	0.723
As	−0.003	0.264	0.592
Cu	0.784	0.113	−0.134
Pb	0.555	0.186	0.381
Zn	0.859	0.145	−0.027
Ni	0.147	0.907	0.075
Cr	0.127	0.901	0.035
Characteristic root	2.131	1.792	1.128
Percentage of characteristic root/%	26.641	22.401	14.105
Cumulative percentage/%	26.641	49.041	63.146

**Table 9:** Boundary of single factor ecological risk level division.

Level	First-Class	Second-Class	Third-Class
Pollution risk	Risk-free	Risk-controllable	High risk
Classification criterion	*C_i_* ≤ *S_i_*	*S_i_* < *C_i_* ≤ *Gi*	*C_i_* > *G_i_*

**Table 10:** Toxicity coefficient (*T_i_*) of heavy metal.

Element	Zn	Cr	Cu	Pb	Ni	As	Cd	Hg
Toxicity coefficient	1	2	5	5	5	10	30	40

**Table 11:** Potential ecological risk assessment criteria.

Single Factor Potential Ecological Risk Coefficient	Potential Ecological Risk Index	Pollution Level	References
*E_i_* < 40	RI < 150	Mild	[44,49]
40 ≤ *E_i_* < 80	150 ≤ RI < 300	Moderate	
80 ≤ *E_i_* < 160	300 ≤ RI < 600	Strong	
160 ≤ *E_i_* < 320	RI ≥ 600	Very strong	
*E_i_* ≥ 320	/	Extremely strong	

**Table 12:** Statistical table of single factor and comprehensive area of heavy metal pollution in topsoil.

Element	Area/km2		
	Risk-Free	Risk-Controllable	High Risk
Pb	1525.64	0.07	/
Zn	1525.15	0.56	/
As	1525.41	0.02	0.28
Hg	1525.67	0.03	0.002
Cu	1519.08	6.62	/
Cd	1522.94	2.30	0.46
Ni	1525.13	0.57	/
Cr	1525.58	0.13	/
**Comprehensive**	**1515.37**	**9.71**	**0.63**

**Table 13:** Single factor potential risk factor of HMs in topsoil.

Single Factor	Potential Ecological Risk Coefficient-*E_i_*		
	Average	Minimum	Maximum
Cd	31.57	6.00	1044.00
Hg	56.96	4.51	14424.06
As	10.15	2.29	382.45
Cu	5.72	0.56	216.81
Pb	5.08	2.15	34.01
Zn	1.08	0.15	38.31
Ni	5.12	0.89	64.47
Cr	2.03	0.58	16.43

## Data Availability

The data presented in this study are available on request from the corresponding author. The data are not publicly available because the project team do not allow us to publish the data of this study.
